# An audit of Individual Care Plan (ICP) in Dublin North City and County (DNCC) child and adolescent mental health service (CAMHS)

**DOI:** 10.1192/bjo.2021.245

**Published:** 2021-06-18

**Authors:** Uchechukwu Egbuta, Cillian Howley, Anitha Selvarajoo, Muhammad Iqbal, Diana Meskauskait

**Affiliations:** Health Service Executive, Ireland

## Abstract

**Aims:**

The objectives/aims of the Audit include:

1. To standardize and implement ICP for service users attending DNCC CAMHS team in accordance with the established policy.
2. To achieve greater involvement of service users/parents in ICP.
3. To standardize and improve treatment of care involving all members of one team.

**Background:**

Every patient should have a care plan. Each care plan has a set of needs and goals. These are agreed between the service user and key worker and are assessed and measured frequently. Consultation with each service user/parents, as far as practicable is important. Specification of treatment and care required in accordance with best practice should be recorded. Identification of the necessary resources should be recorded and discussed with service user and key worker. Records kept in one composite set of documentation, and a signed copy should be made available to the service user/parents.

**Method:**

First Cycle commenced 15th October 2019. 166 files were selected from CAMHS team. Data were collected from clinical records from time of admission into CAMHS service to the time of audit. The audit report was prepared on the 6th December 2019, and intervention discussed at the multidisciplinary team meeting and wider DNCC CAMHS academic meeting. Second Cycle 23rd March 2020. 30 files randomly selected and audited. Data were collected by Dr Uchechukwu Egbuta, Mr Cillian Howley, Dr Anitha Selvarajoo, under supervision of Dr Muhammad Iqbal and Dr Diana Meskauskaite.

Method of data input/analysis is IBM SPSS.

**Result:**

For each ICP, the following were looked at: Files with ICP, Identifiable key worker, Formulation, Goals, Action plan, Copy of ICP to young person/parents, Next Review Date, Projected discharge date.

Overall compliance shows 62% in first cycle, and 68% in second cycle after intervention.

There was a 6% quality improvement of ICPs in terms of overall compliance in applying the various components of ICP.

**Conclusion:**

Each service user should have an individual care plan. Each individual care plan should be measured regularly. To develop a therapeutic individual care plan, a formulation of the case from history taking is essential looking at the bio-psychosocial model and should be service user focused. Care plans are part of clinical governance, therefore continuous re-audit every three months was recommended. The follow-up audit will be carried out by the multidisciplinary team members.

